# Purifying Selection Determines the Short-Term Time Dependency of Evolutionary Rates in SARS-CoV-2 and pH1N1 Influenza

**DOI:** 10.1093/molbev/msac009

**Published:** 2022-01-17

**Authors:** Mahan Ghafari, Louis du Plessis, Jayna Raghwani, Samir Bhatt, Bo Xu, Oliver G Pybus, Aris Katzourakis

**Affiliations:** 1 Department of Zoology, University of Oxford, Oxford, United Kingdom; 2 MRC Centre for Global Infectious Disease Analysis, Jameel Institute for Disease and Emergency Analytics, Imperial College London, London, United Kingdom; 3 Department of Earth System Science, Tsinghua University, Beijing, China

**Keywords:** substitution rate, molecular clock, clock rate, purifying selection

## Abstract

High-throughput sequencing enables rapid genome sequencing during infectious disease outbreaks and provides an opportunity to quantify the evolutionary dynamics of pathogens in near real-time. One difficulty of undertaking evolutionary analyses over short timescales is the dependency of the inferred evolutionary parameters on the timespan of observation. Crucially, there are an increasing number of molecular clock analyses using external evolutionary rate priors to infer evolutionary parameters. However, it is not clear which rate prior is appropriate for a given time window of observation due to the time-dependent nature of evolutionary rate estimates. Here, we characterize the molecular evolutionary dynamics of SARS-CoV-2 and 2009 pandemic H1N1 (pH1N1) influenza during the first 12 months of their respective pandemics. We use Bayesian phylogenetic methods to estimate the dates of emergence, evolutionary rates, and growth rates of SARS-CoV-2 and pH1N1 over time and investigate how varying sampling window and data set sizes affect the accuracy of parameter estimation. We further use a generalized McDonald–Kreitman test to estimate the number of segregating nonneutral sites over time. We find that the inferred evolutionary parameters for both pandemics are time dependent, and that the inferred rates of SARS-CoV-2 and pH1N1 decline by ∼50% and ∼100%, respectively, over the course of 1 year. After at least 4 months since the start of sequence sampling, inferred growth rates and emergence dates remain relatively stable and can be inferred reliably using a logistic growth coalescent model. We show that the time dependency of the mean substitution rate is due to elevated substitution rates at terminal branches which are 2–4 times higher than those of internal branches for both viruses. The elevated rate at terminal branches is strongly correlated with an increasing number of segregating nonneutral sites, demonstrating the role of purifying selection in generating the time dependency of evolutionary parameters during pandemics.

## Introduction

Rapid whole-genome sequencing has become part of pathogen surveillance systems and is important to both infection control and enables a detailed investigation of the epidemiological and evolutionary characteristics of pathogens. Quantifying infectious disease evolution enables the inference of parameters such as times of origin, epidemic growth rates, and evolutionary rates (Fraser et al. 2009; Smith et al. 2009; Lu et al. 2020).

One of the perils of making such inferences over short time periods (i.e., a few months or years) is that the inferred parameters of interest may vary over the timespan of observation (Meyer et al. 2015). Although several factors can lead to the misestimation of substitution rates (such as misidentification of coalescent population models, changes in natural selection, differing replication rates in various host reservoirs, or sequencing errors), misspecification of clock models and saturation of nucleotide changes can result in rate underestimation (Clark and Whittam 1992; Sullivan and Joyce 2005; Duchêne et al. 2014; Ghafari et al. 2021). This nonstationarity can render findings that are confusing or conflicting such that an estimated substitution rate over one time window is not transferable to the analysis of another (Ho et al. 2011). For example, during the 2014–2016 West Africa Ebolavirus epidemic, there was controversy and concern regarding the virus’ “mutation rate,” because early estimates of the substitution rate from the epidemic appeared to be approximately twice the average rate across multiple outbreaks (Gire et al. 2014; Holmes et al. 2016). Confusion such as this is also common in the SARS-CoV-2 literature and arises in part due to an incomplete understanding of estimated substitution rates by users of phylogenetic analysis software. Given the importance of phylogenetic dating and clock estimation for all SARS-CoV-2 genomic epidemiology worldwide, understanding the pattern of inferred substitution rate over time and investigating the potential underlying mechanisms involved in creating time-dependent rate effects in viruses can shed light on the molecular evolutionary dynamics of viruses.

Another major obstacle is that during an ongoing outbreak, the level of sequence diversity may be so low that the amount of accrued evolutionary change is not sufficient to make informative phylogenetic inference possible (Duchêne et al. 2020). Several statistical tests have been developed to ensure enough temporal signal is present in a set of temporally sampled genome sequences to reliably infer evolutionary parameters (Duchêne et al. 2015; Murray et al. 2016; Duchêne S and Duchêne DA 2020).

More specifically, there is extensive evidence that purifying selection in viruses can result in varied estimates for rates of substitution and evolutionary rate ratio, d*N*/d*S*, across different timescales (Sharp et al. 2001; Holmes 2003; Hughes and Hughes 2007). It is likely that a greater proportion of polymorphisms observed among genomic sequences sampled early in an epidemic are segregating deleterious mutations, which will only persist for a limited time before being eliminated by purifying selection (Lam et al. 2016). The duration of this time-dependent effect may be prolonged due to incomplete purifying selection in populations with very large effective population sizes (Woodhams 2006).

Although evidence of strong purifying selection has mostly been reported at the species level and over very long timescales (i.e., thousands to millions of years), using inference methods based on the d*N*/d*S* ratio (Ho et al. 2005; Pybus et al. 2007; Wertheim and Pond 2011), there have been few studies at the intra-population level and over short timescales, mainly because there is often no opportunity to collect sufficiently large numbers of samples through time to track low-frequency variants (Hedge et al. 2013; Meyer et al. 2015). Crucially, although purifying selection has been put forward as a possible explanation for the time dependency of substitution rates over such timescales, there have not been any systematic studies to investigate the role of purifying selection and quantify its contribution to altering the inferred substitution rate of viruses over time. Furthermore, using standard phylogenetic methods to compute the d*N*/d*S* ratio for conspecific sequences sampled from a single population over short timescales may be inappropriate as the differences between sequences over such timescales represent segregating polymorphisms as opposed to fixed substitutions along independent lineages. The former has been shown to produce very different estimates of the d*N*/d*S* ratio over time (Rocha et al. 2006; Kryazhimskiy and Plotkin 2008).

In this study, we aim to identify and quantify the source of time dependency of virus substitution rate estimates over short time periods and characterize estimates of the molecular clock rate, time of origin, growth rate, and number of nonneutral sites for different data sets that represent different timescales of genomic observation. We show that time dependency in estimates of the mean substitution rate is dominated by elevated rates at terminal branches, whereas the estimated rate of substitution at internal branches does not exhibit a time-dependent decay. We then use a generalized McDonald–Kreitman test based on nucleotide site frequencies that allows purifying selection to be quantified over short timescales and demonstrate that there is a strong correlation between the elevated rates at terminal branches and the high number of low frequency nonneutral sites in both SARS-CoV-2 and pH1N1 genomes.

## Results

**Fig. 1.**
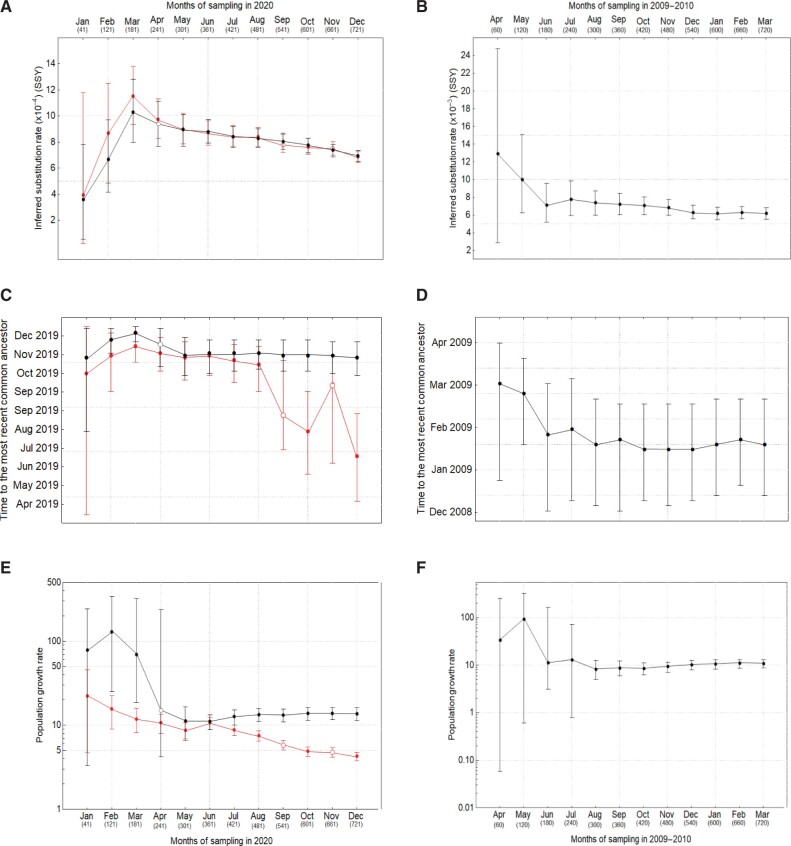
(A, *B*) Inferred rates, (*C*, *D*) times of origin, and (*E*, *F*) growth rates of SARS-CoV-2 (left column) and pH1N1 influenza (right column) using an exponential (red) and a logistic (black) growth model. Open circles represent nonconvergence for at least one parameter in the Bayesian analysis. Note that y-axes are not on the same scale for SARS-CoV-2 and pH1N1.

We use Bayesian phylogenetic methods implemented in BEAST v.1.10 to estimate the molecular clock rate, times of origins, and growth rates of the SARS-CoV-2 and pH1N1 pandemics. The data sets varied in size and temporal sampling range and were created to reflect the way in which real data sets accumulate in size and diversity during the course of an epidemic. Specifically, we aggregate all available samples up to each respective month and infer the parameters of interest (fig. 1 and supplementary fig. S1, Supplementary Material online). We first compare the results for SARS-CoV-2 using two coalescent growth priors: exponential growth and logistic growth. Although this is not a comprehensive comparison between all phylogenetic models that can be used to explain the evolutionary dynamics, it allows us to find the better-fitting model that introduces less error in parameter estimation—the demographic model is effectively a nuisance variable. Our analysis suggests, except for the month of January, the logistic growth coalescent tree prior is a better fit to the data (table 1). As reported in previous studies, we find that there is not enough temporal signal in the data to reliably infer the evolutionary parameters during the first 2 months of the SARS-CoV-2 pandemic (Duchêne et al. 2020). This results in the underestimation of substitution rates as well as high statistical uncertainty for the parameter estimates. The lack of temporal signal in the SARS-CoV-2 samples is also evident from the number of new singletons (i.e., single nucleotide variations in the data set compared with the ancestral sequence) added the data set per month during the first 2–3 months (supplementary fig. S2, Supplementary Material online). On the other hand, for pandemic H1N1 (pH1N1), the molecular clock rate is up to two times higher for the first 3 months than for the following months (fig. 1*A* and *B*). Although the inferred substitution rate of SARS-CoV-2 tends to decrease as we increase the timespan of measurement, the rate for pH1N1 does not change considerably after the first 3–4 months of measurement, in agreement with previous findings (Meyer et al. 2015).

**Table 1.** Log-Marginal Likelihoods of Exponential and Logistic Growth Models with Increasing Temporal Ranges of Sampling Dates.

Month of Sampling	Number of Samples	Log-Marginal Likelihood Exponential Growth Model	Log-Marginal Likelihood Logistic Growth Model	Bayes Factor
Jan	41	−41,022.77	−41,025.56	−2.79
Feb	121	−43,062.00	−43,047.11	+14.88
Mar	181	−44,627.77	−44,607.95	+19.78
Apr	241	−46,524.75	−46,506.25	+18.53
May	301	−48,587.39	−48,548.15	+39.24
Jun	361	−52,310.61	−52,248.23	+62.38
Jul	421	−55,138.43	−55,036.60	+101.83
Aug	481	−58,580.94	−58,430.09	+150.85
Sep	541	−62,101.22	−61,805.93	+295.29
Oct	601	−65,433.20	−65,005.09	+428.11
Nov	661	−69,121.03	−68,921.98	+199.05
Dec	721	−73,094.26	−72,389.69	+704.57

Note.—Taking exponential growth as the null model, we select the logistic growth model for any data set with a positive Bayes factor.

Both the inferred times of origin and growth rates of SARS-CoV-2 and pH1N1 in early months have wide credible intervals due to the high uncertainty associated with small sample sizes and narrow sampling windows. The precision of these two inferred parameters increases with the addition of data from later months and remains roughly consistent after the first 4–5 months of measurement (fig. 1*C*–*F*). Using the logistic growth model, we find the estimated times of origin for SARS-CoV-2 and pH1N1 to be October 28, 2019 (95% HPD: September 30, 2019, November 24, 2019) and January 18, 2009 (95% HPD: December 14, 2008, February 22, 2009), respectively, which is also in agreement with previous studies (Smith et al. 2009; Hedgeet al. 2013; Lu et al. 2020). However, under the exponential growth model, the inferred time of origin of SARS-CoV-2 samples significantly diverge from the expectation after 6 months of sampling and yield unreliable estimates (fig. 1*C* and *D*). This is likely because after the first few months, the growth rate declines and the population dynamics deviate from the exponential growth model (fig. 1*E* and *F*). This, in turn, results in the underestimation of growth rate of SARS-CoV-2 and inferring an older time of origin. We also note that, from a molecular epidemiology perspective, using the exponential growth coalescent model would not be appropriate nor realistic particularly over longer timescales (i.e., >6 months) as the pandemic dynamics did not continue to grow exponentially (i.e., the growth rate started to drop after the first few months due to various behavioral changes in the population and the implementation of nonpharmaceutical interventions). Despite the difference in the estimated time of origin, both the exponential and logistic growth models infer very similar clock rates for SARS-CoV-2 over time.

**Fig. 2.**
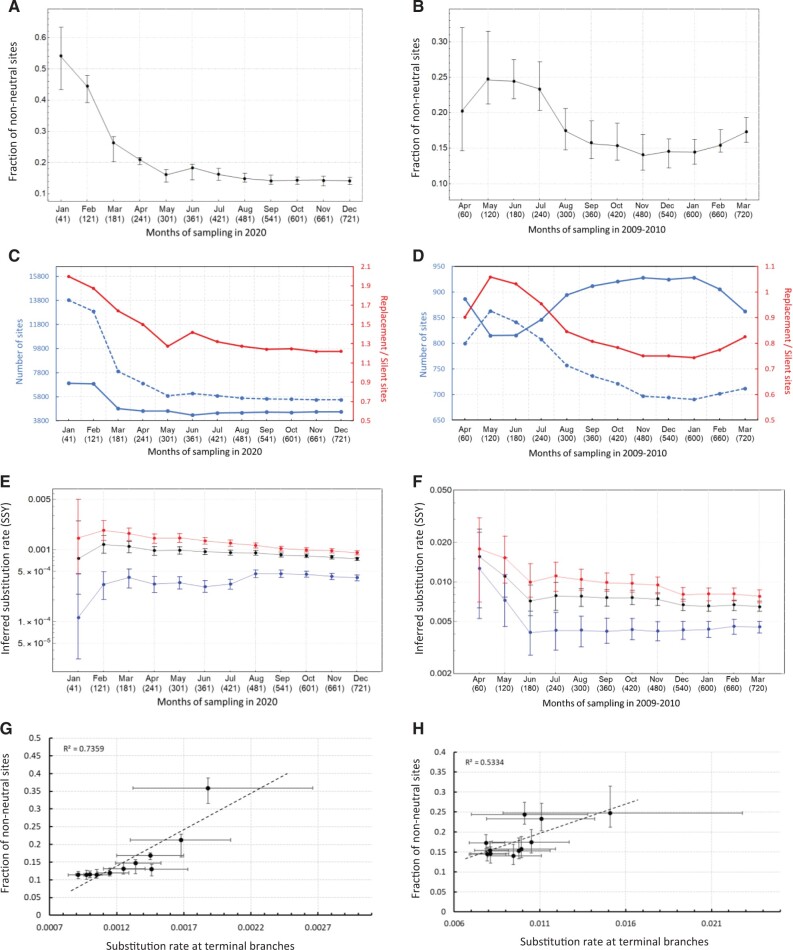
(A, *B*) Number of nonneutral sites over time for SARS-CoV-2 (left column) and pH1N1 (right column). (*C*, *D*) Number of replacement (dashed blue line), silent (solid blue line) sites, and their ratio (red line) over time. (*E*, *F*) Mean clock rate (black), and the rates at the terminal (red) and internal (blue) branches. The MCMC chains for the first month of sampling SARS-CoV-2 and the first three months of pH1N1 do not converge using the logistic growth coalescent model. Instead, the exponential growth coalescent model was used. (*G*, *H*) Correlation coefficient between the substitution rate at terminal branches and number of nonneutral sites—excluding the estimates from the first month of sampling due to inadequate temporal signal and significant uncertainty in the inferred parameters.

Purifying selection has been often cited as one of the main evolutionary processes contributing to the elevation of estimated molecular evolutionary rates over short timescales (Hedge et al. 2013). The argument is that low-frequency deleterious mutations can segregate in a population for some time before being purged because of purifying selection. Therefore, the proportion of all changes that are deleterious is high when the phylogenetic tree is short, and lower when the tree is longer as it takes more time for shared neutral or advantageous changes to accrue with respect to the ancestral state. To evaluate this hypothesis, we investigate the correlation between the number of nonneutral polymorphisms and estimated substitution rates over time. Our results show that there is a higher proportion of segregating mutations at low frequencies (<15%) during the first few months of observation, in both the ORF1ab of SARS-CoV-2 and the HA of pH1N1 influenza (fig. 2*A* and *B*). In particular, the proportion of low-frequency nonneutral sites in ORF1ab was higher during the first 4–5 months of observation and had a roughly 4-fold drop during that period compared with a 2-fold drop in HA gene. After the first 5 months, the proportion of low frequency nonneutral sites remains roughly constant at around 15% in ORF1ab, whereas it shows an uptick from 15% to 17% in HA toward the beginning of 2010. The slight increase in the proportion of nonneutral sites in HA during this period is also in agreement with a rise in the relative genetic diversity of pH1N1 around the world in late 2009/early 2010 which may also have resulted in an increase in the number of segregating deleterious mutations in the population (Su et al. 2015). We note that although it is unlikely for mutations in the low frequency class to have strongly positive fitness effects, they may also contain neutral or adaptive mutations that have not reached sufficiently high frequencies yet. We also find that the number of low frequency replacement sites is always greater than silent sites in ORF1ab whereas, for HA, silent sites are in majority in most months (fig. 2*C* and *D*). The total number of nonneutral sites in ORF1ab is higher because it is a much longer gene (∼21,000 nt) compared with HA (∼1,800 nt).

One of the impacts of purifying selection over short timescales is that the number of replacements on terminal branches should be higher than that on internal branches. To measure this effect during the SARS-CoV-2 and pH1N1 epidemics, we used a branch-specific two-parameter molecular clock model in BEAST and infer separate rates of substitution for terminal and internal branches (see fig. 2*E* and *F*). Our results show that for both viruses, the rates of substitution on terminal branches are 2–4 times higher than on internal branches. The difference is more dramatic in SARS-CoV-2 where the average ratio of substitution rates at terminal branches are four times higher than that of internal branches whereas it is only two times higher in HA during the first 12 month of observation. Figure 2*E* and *F* also shows that the mean and credible intervals of the inferred substitution rate at terminal and internal branches are nonoverlapping during several months of rate measurement which demonstrates that there is a significant difference between the rate of evolution at these two branch categories. This also agrees with the observation of a higher proportion of nonneutral sites in SARS-CoV-2 samples compared with pH1N1 because there is going to be more deleterious mutations at terminal branches of SARS-CoV-2. Further, there is a clear decline in the estimated rate at terminal branches as the sampling window increases. In contrast, the estimated rate at internal branches gradually increases through time (with the exception of the first 2–3 months for pH1N1).

We also investigate the role of sequencing error as a potential confounder for the elevated rates at terminal branches. By masking more than 110 sites of SARS-CoV-2, in addition to our standard filtering step (see Materials and Methods section), which are suggested to be prone to recurrent sequencing error and appear to be highly homoplastic, we recalculate the rate of evolution at terminal and internal branches (supplementary fig. S3, Supplementary Material online). We find that although the overall inferred rates drop for the alignments with masked sites (due to their lower genetic variation compared with unmasked alignments), the time-dependent rate drop at terminal branches is retained (supplementary fig. S3*B*, Supplementary Material online). Therefore, sequencing error cannot be the underlying source for time dependency of evolutionary rates. In figure 2*G* and *H*, we further show that there is an overall significant correlation between the rate of substitution at terminal branches for the unmasked alignments and number of nonneutral sites in both SARS-Cov-2 (*R*^2^ = 0.74 and *P* < 0.001) and pH1N1 (*R*^2^ = 0.53 and *P* < 0.01) (fig. 2*G* and *H*). By examining the site frequency spectrum for both data sets, we can also see that most of the variation comes from the low-frequency regime (<15%) with very limited variation in the mid- to high-frequency regimes (supplementary fig. S2, Supplementary Material online).

Furthermore, in the pH1N1 data set, we can see a dip in the inferred rate at terminal branches in December 2009 (fig. 2*F*). There is also a sudden loss of low frequency genetic diversity in the data set during the same period which is partially recovered in February and March (see the trajectory of newly added singletons and low frequency variants in supplementary fig. S2*D* and *F*, Supplementary Material online). On the other hand, the pattern of substitution rate at internal branches is nonmonotonic over time. In particular, we see an increase in the rate at internal branches of SARS-CoV-2 from August onward. Similarly, there is a steady rise in the rate at internal branches of pH1N1 from February onward.

## Discussion

We found that the overabundance of deleterious mutations during the early stages of both the SARS-CoV-2 and pH1N1 influenza pandemics strongly correlates with higher substitution rates at terminal branches relative to internal branches of inferred phylogenetic trees. Once there is enough temporal signal in the data to reliably estimate relevant epidemiological and evolutionary parameters, the mean substitution rates decline over the course of 1 year of rate measurement, whereas the estimated time of origin and growth rate are more stable and remain roughly the same after the first 4–5 months of measurement. We found that this declining pattern in mean substitution rate is caused by the rate decay at terminal branches whereas the rate at internal branches does not exhibit such a pattern over time. In particular, the gradual increase in the substitution rate at internal branches over the span of several months for both SARS-CoV-2 and pH1N1 influenza could be related to signatures of adaptive evolution. We also found that sites that are prone to recurrent sequencing error and appear to be highly homoplastic do not make a significant contribution to the time-dependent pattern of substitution rates. Although both sequencing error and deleterious mutations appear as singletons or low-frequency variations in the data, deleterious mutations are strongly biased toward changing amino acids, whereas sequencing errors are equally likely to be synonymous or nonsynonymous. Therefore, they do not make similar contributions to altering the inferred substitution rates over time.

Our results provided further evidence that short-term rate estimates are subject to time-dependent rate effects largely due to transient polymorphisms (Hedgeet al. 2013; Meyer et al. 2015). In addition, our estimated mean substitution rates, times of origin, and growth rates agree with previous studies of SARS-CoV-2 (Lu et al. 2020; Volz et al. 2020; Duchêne et al. 2020; du Plessis et al. 2021) and pH1N1 influenza (Rambaut and Holmes 2009; Smith et al. 2009; Hedge et al. 2013; Meyer et al. 2015). We found that although the time-dependent rate effect for pH1N1 is less pronounced after the first 3 months of sampling, the same effect in SARS-CoV-2 is more pronounced and continues even after 12 months of sampling. This may be the result of a much higher mean substitution rate in pH1N1 compared with SARS-CoV-2. We also found a higher proportion of low frequency nonneutral sites for the SARS-CoV-2 data set during the first 8 months of observations and a higher ratio of replacement to silent sites during the entire 1 year of observation compared with pH1N1. The same method used in this study to infer the number of low frequency nonneutral sites has also been used to characterize the molecular evolutionary dynamics of other RNA viruses including seasonal coronaviruses and emerging avian influenza viruses (Bhatt et al. 2013; Lu et al. 2018; Kistler and Bedford 2021).

By dividing the data into successively longer temporal intervals, we showed that the overabundance of deleterious mutations at terminal branches is the main reason behind the gradual decay in mean substitution rate over time in SARS-CoV-2 and pH1N1 influenza. Further work can be done to quantify the longer-term effect of purifying selection on the time dependency of substitution rates.

## Materials and Methods

We downloaded all SARS-CoV-2 sequences from GISAID and pH1N1 influenza sequences from GenBank and aligned them using MUSCLE v3.8.425 (Edgar 2004)—a complete metadata table acknowledging the authors, originating and submitting laboratories of the SARS-CoV-2 sequence data is available in supplementary table S1, Supplementary Material online. For the SARS-CoV-2 data set, we mask the first 54 and last 240 sites of the alignments and only include complete sequences that are more than 29,000 nt long with high coverage as determined by GISAID’s default search option (i.e., entries with <1% Ns and <0.05% unique amino acid substitutions). Furthermore, for part of the analysis where we investigate the role of sequencing error in time-dependent rate effects at terminal branches for SARS-CoV-2, we mask an additional 114 sites that appear to be highly homoplastic or prone to recurrent sequencing error (De Maio et al. 2020, https://virological.org/t/issues-with-sars-cov-2-sequencing-data/473). We specifically select pH1N1 sequences from GenBank for the full coding region of the hemagglutinin (HA) and ensure that none of the SARS-CoV-2 and pH1N1 samples have undergone serial passaging.

To investigate the effects of the temporal range of sampling dates on the accuracy of parameter estimation, we incrementally increase the size of each data set by adding 60 genomes, chosen randomly, for every additional month of sampling. Thus, after 12 months of sampling since the first sequence was uploaded on GISAID and/or GenBank, we have 720 samples. We note that due to a lack of temporal signal in the early SARS-CoV-2 samples (i.e., most of the early samples were almost completely identical) and failure of the Markov chain Monte Carlo (MCMC) chains for the phylogenetic analyses to converge, we only used 41 samples collected between December 24, 2019 and January 31, 2020 (labeled as “January sequences” in our analysis) and took 20 additional samples during the next month to match with the 120 samples used for pH1N1 by the end of the second month of sampling (see supplementary fig. S1, Supplementary Material online).

### Phylogenetic Analyses

We use BEAST v1.10 (Suchard et al. 2018) for the Bayesian phylogenetic analysis of the entire data set using an HKY + Γ substitution model with a Laplace prior (mean = 0 and scale = 100) on the coalescent growth rate, a Lognormal prior (mean = 1 and SD = 2) on the coalescent population size, and a continuous time Markov chain prior on the evolutionary clock rate. For the first part of the analysis, we use a strict clock model and exponential and logistic growth coalescent demographic models of SARS-CoV-2 evolution and only the logistic growth model for the HA segment of pH1N1. To quantify the relative fit of the two coalescent models for SARS-CoV-2, we compute their log marginal likelihoods using the generalized stepping-stone sampling method and compare their Bayes factors (Fan et al. 2011; Baele and Lemey 2014; Baele et al. 2016). We note that although the growth coefficient of the two coalescent models is not expected to converge to the same value (as they correspond to intrinsically different population dynamics), the two models are formally nested, and the likelihood function of the exponential model exists as a limit of the likelihood function of the logistic model. Therefore, even though the growth rate is effectively a nuisance variable for our purposes, the likelihoods of the two models can be compared via a likelihood ratio test to select the better-fitting one (Pybus and Rambaut 2002).

For each data set, we perform MCMC runs for 100 million steps, sample trees every 10,000 steps, and remove the first 10% of the steps as burn-in. We ensure that the effective sample size for every parameter of interest is >200 using Tracer v1.7 (Rambaut et al. 2018). For the second part of the analysis, we use a two-parameter molecular clock model with one strict clock rate for terminal branches and one strict clock for internal branches, using the same priors as before. This molecular clock model is a version of a fixed local clock whereby rather than having a single global rate on all branches, the terminal and internal branches are allowed to evolve according to different evolutionary rates whereas rate constancy is assumed along the respective branches (Yoder and Yang 2000; Drummond and Suchard 2010). We use an MCMC chain of length 50 million steps, sampling every 1,000 steps and evaluate sampling of the parameters of interest using Tracer v1.7.

### Estimating the Site Frequency and Number of Nonneutral Sites

We use the adapt-a-rate package (Bhatt et al. 2010, 2011; Raghwani et al. 2016), a generalized McDonald–Kreitman test, to estimate the number of nonneutral sites by assuming that deleterious mutations are mostly confined to the low frequency range (0–15%), neutral mutations to the mid frequency range (15–75%), and adaptive mutations to the high frequency range (75–100%). We then estimate the site frequency spectra by comparing the main alignments to an ancestral sequence. For SARS-CoV-2, the ancestral sequence is the earliest sample collected from Wuhan, Wuhan/IPBCAMS-WH-01/2019, and for pH1N1 it is the earliest sample from Mexico, ACQ99614|A/Mexico/4108/2009. The choice of the cut-off frequencies is based on the diffusion approximation of allele frequencies whereby, at equilibrium, most deleterious and adaptive mutations are confined to frequencies <15% and >75%, respectively (Bhatt et al. 2011). It has further been shown that the exact choice of the cut-off frequency for the three frequency classes does not significantly change the estimated number of nonneutral sites (Bhatt et al. 2011).

We use the ORF1ab of SARS-CoV-2 and HA of pH1N1 influenza for this analysis. We also carried out a similar analysis for the other genes of SARS-CoV-2, including the S gene. However, because of the limited genetic diversity present in the sequences and their relatively short size, our generalized McDonald–Kreitman test using adapt-a-rate is unable to estimate the number of nonneutral sites. The reason for choosing to analyze the HA is primarily because of the availability of a large number of sequences from GenBank for the full coding region of HA which allows us to randomly sample 60 alignments per month from April 2009 to March 2010.

## Supplementary Material

Supplementary data are available at *Molecular Biology and Evolution* online.

## Supplementary Material

msac009_Supplementary_Data
