# Supplementary material for: Purifying Selection Determines the Short-Term Time Dependency of Evolutionary Rates in SARS-CoV-2 and pH1N1 Influenza
Source: Mol Biol Evol. 2022 Jan 17;39(2):msac009. doi: 10.1093/molbev/msac009 (PMC8826518; doi:10.1093/molbev/msac009)
Supplement: msac009_Supplementary_Data [file msac009_supplementary_data.zip › Supplementary Figures final MBE.docx]

# **Supplementary Figures**

| **(A)** | **(B)** |
| --- | --- |
| 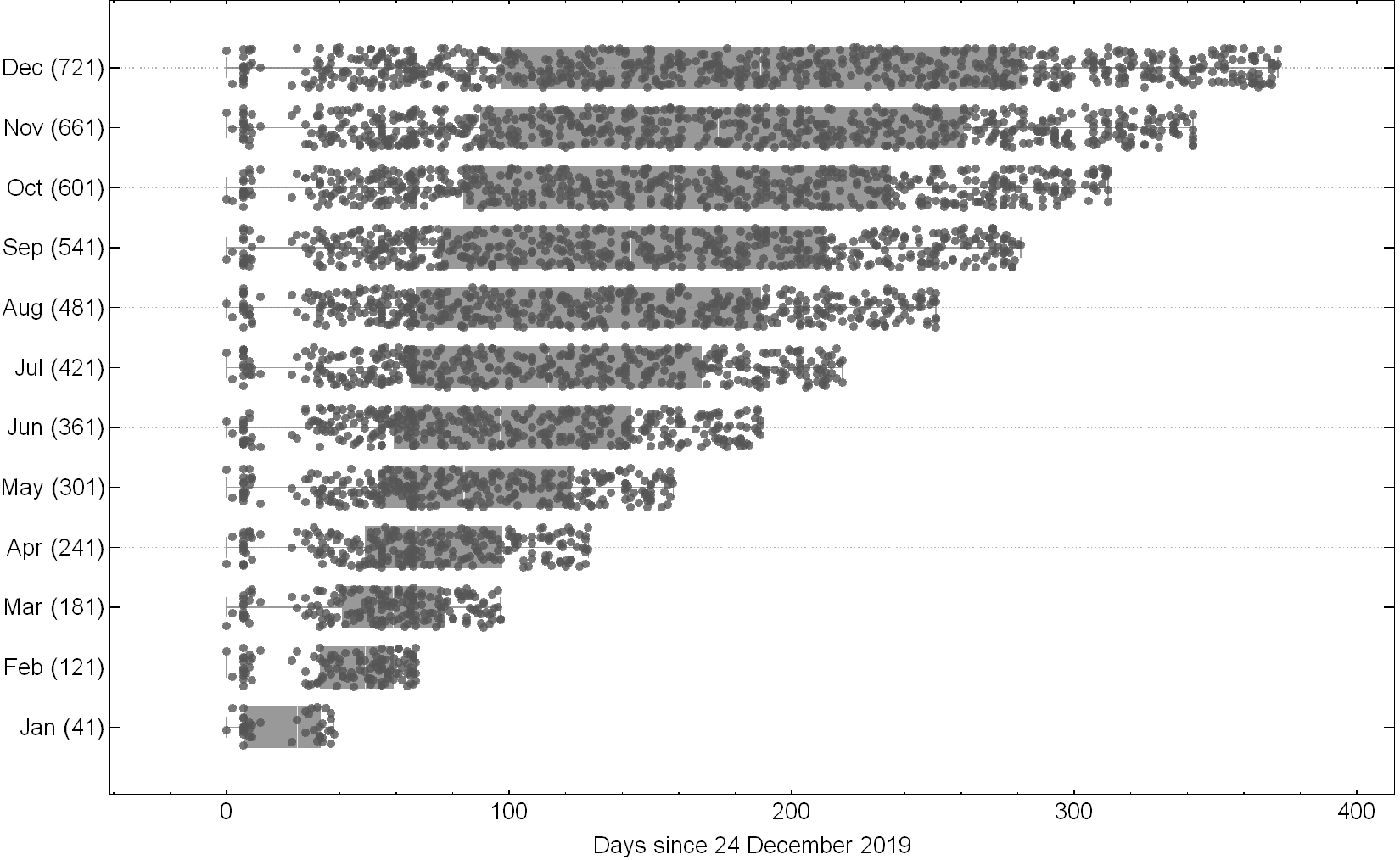 | 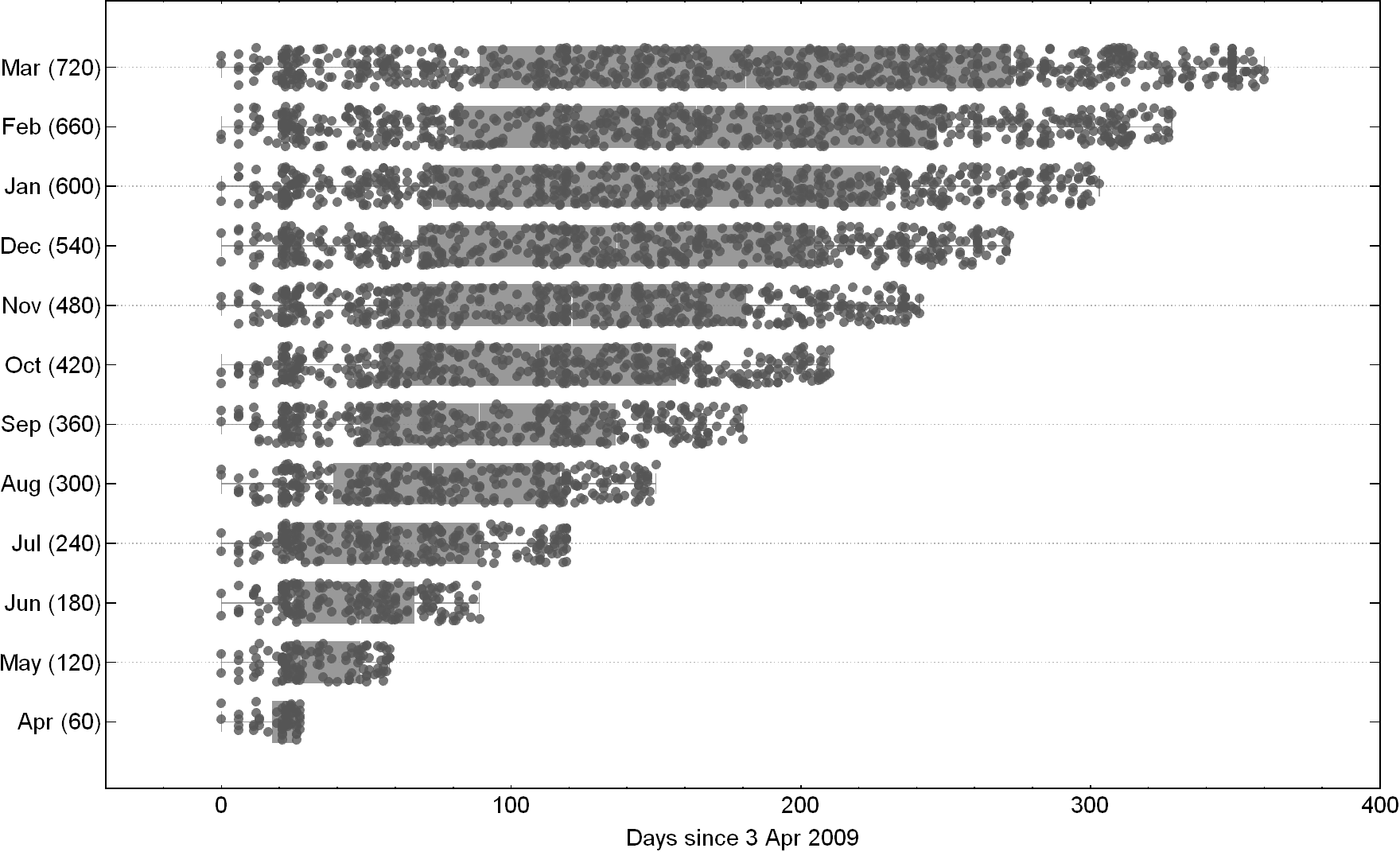 |
| **Figure S1:** Sampling intervals and numbers of sequences in the different datasets used for SARS-CoV-2 and pH1N1 analyses. The sampling interval is measured from the day when the first genome was collected. | |

| **(A)** | **(B)** |
| --- | --- |
| 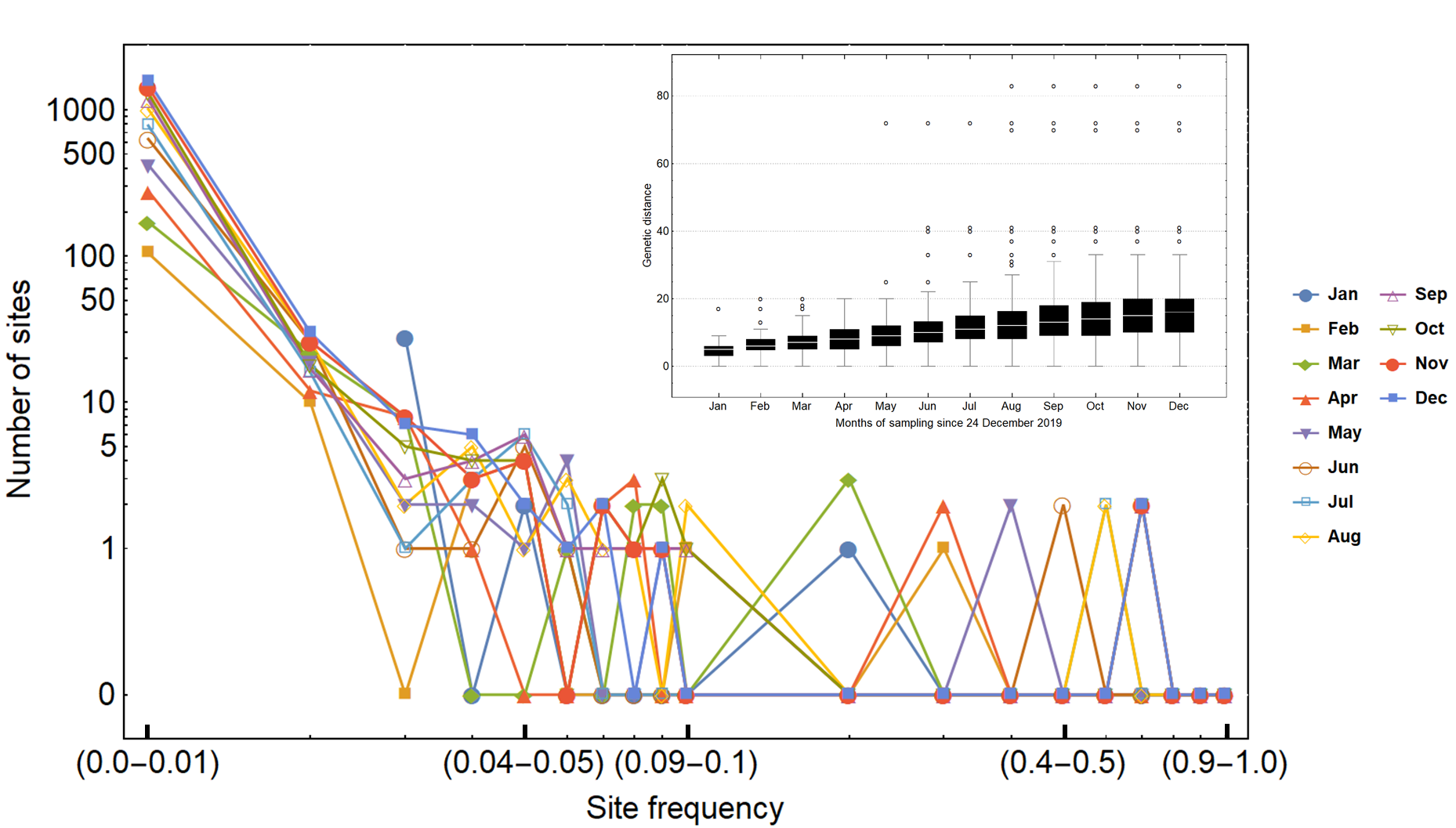 | 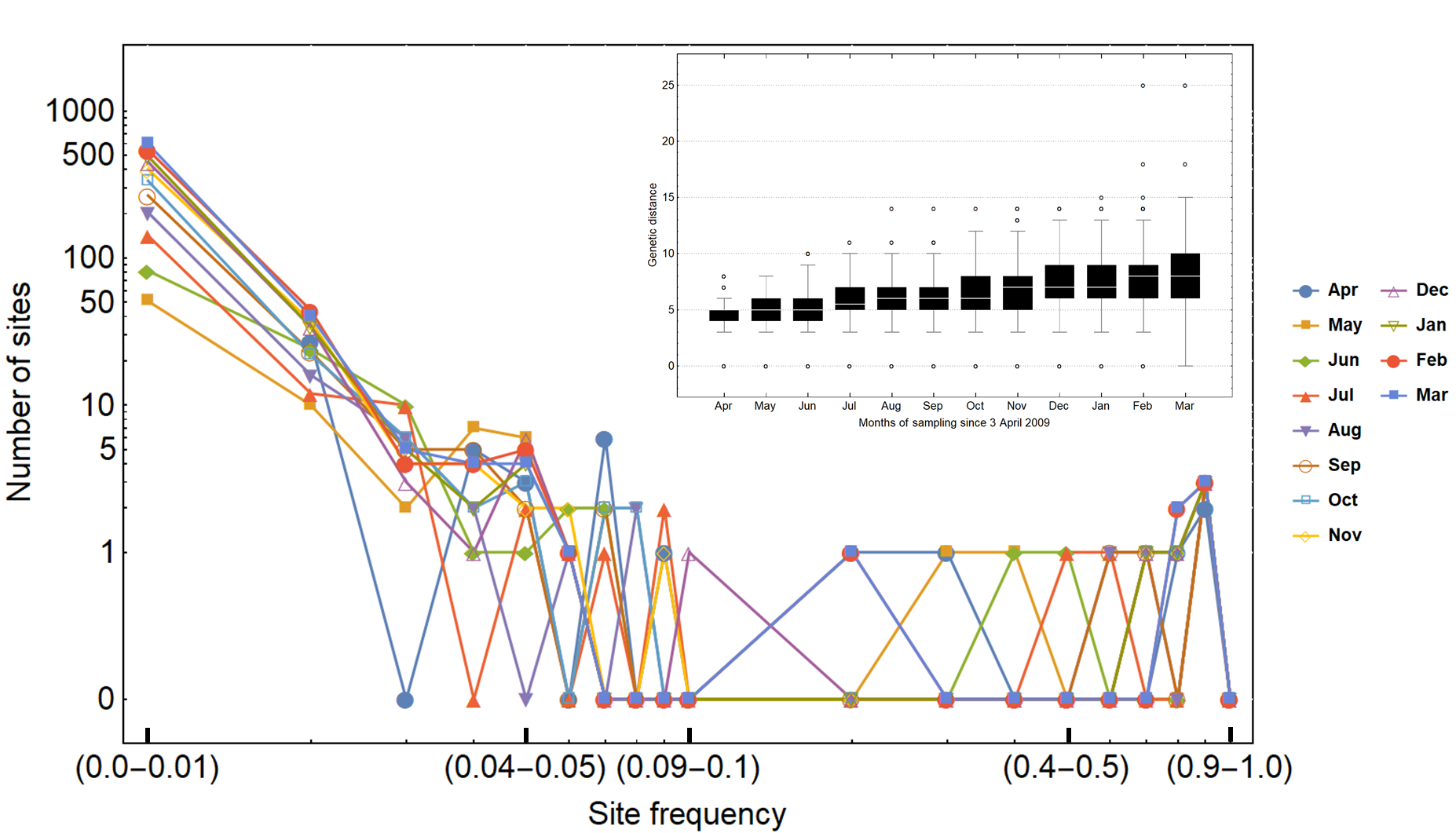 |
| **(C)** | **(D)** |
| 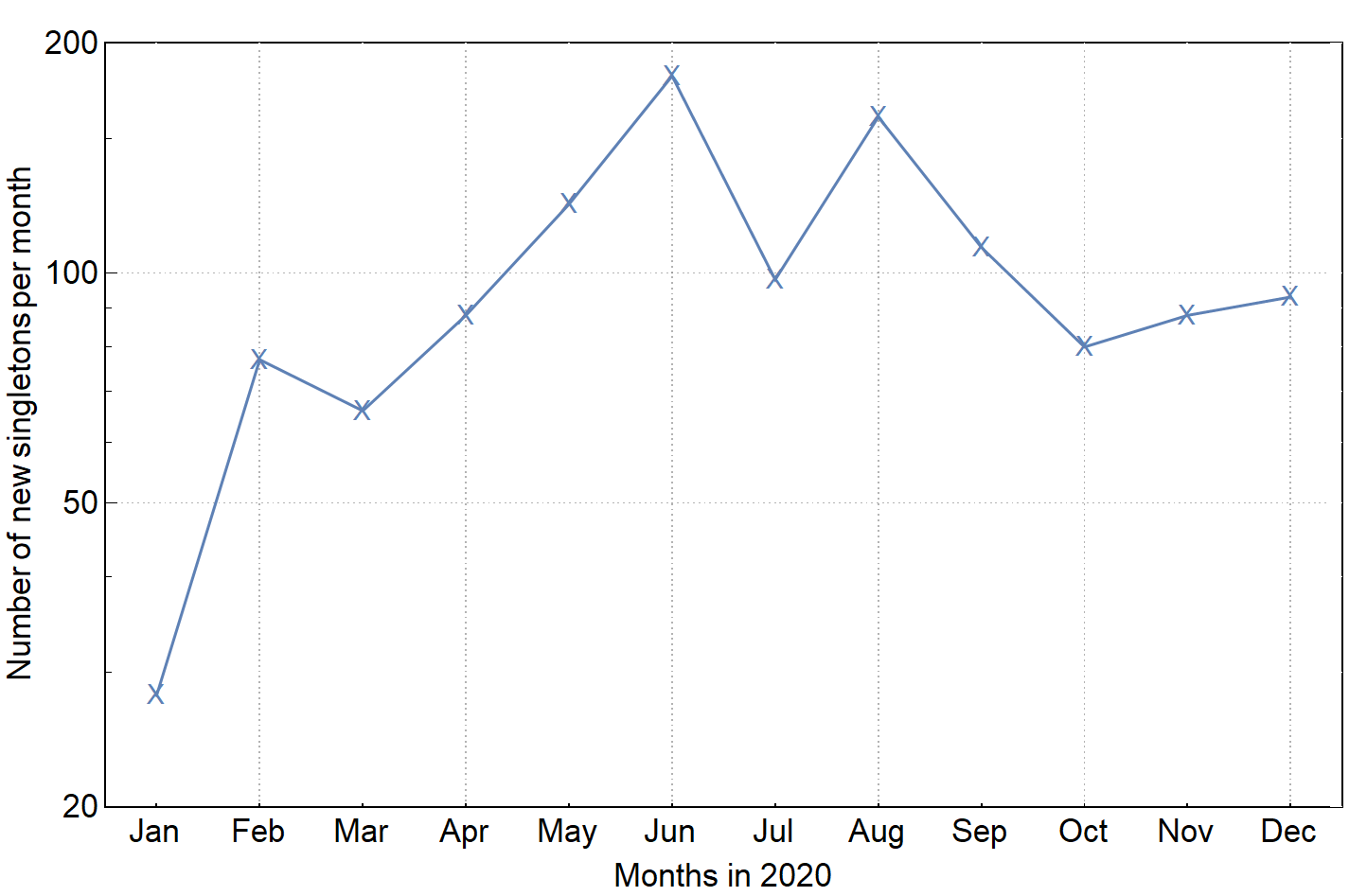 | 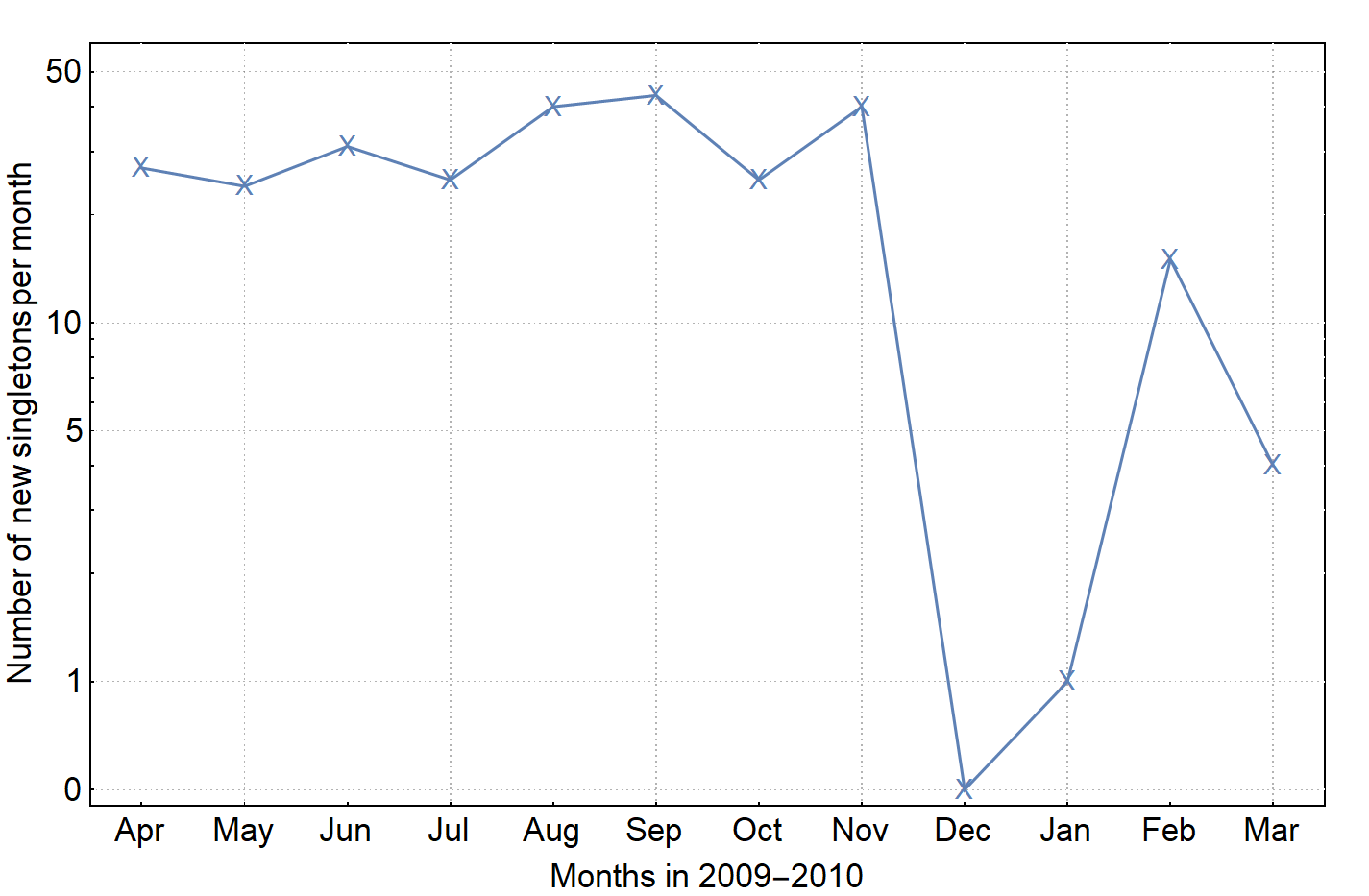 |
| **(E)** | **(F)** |
| 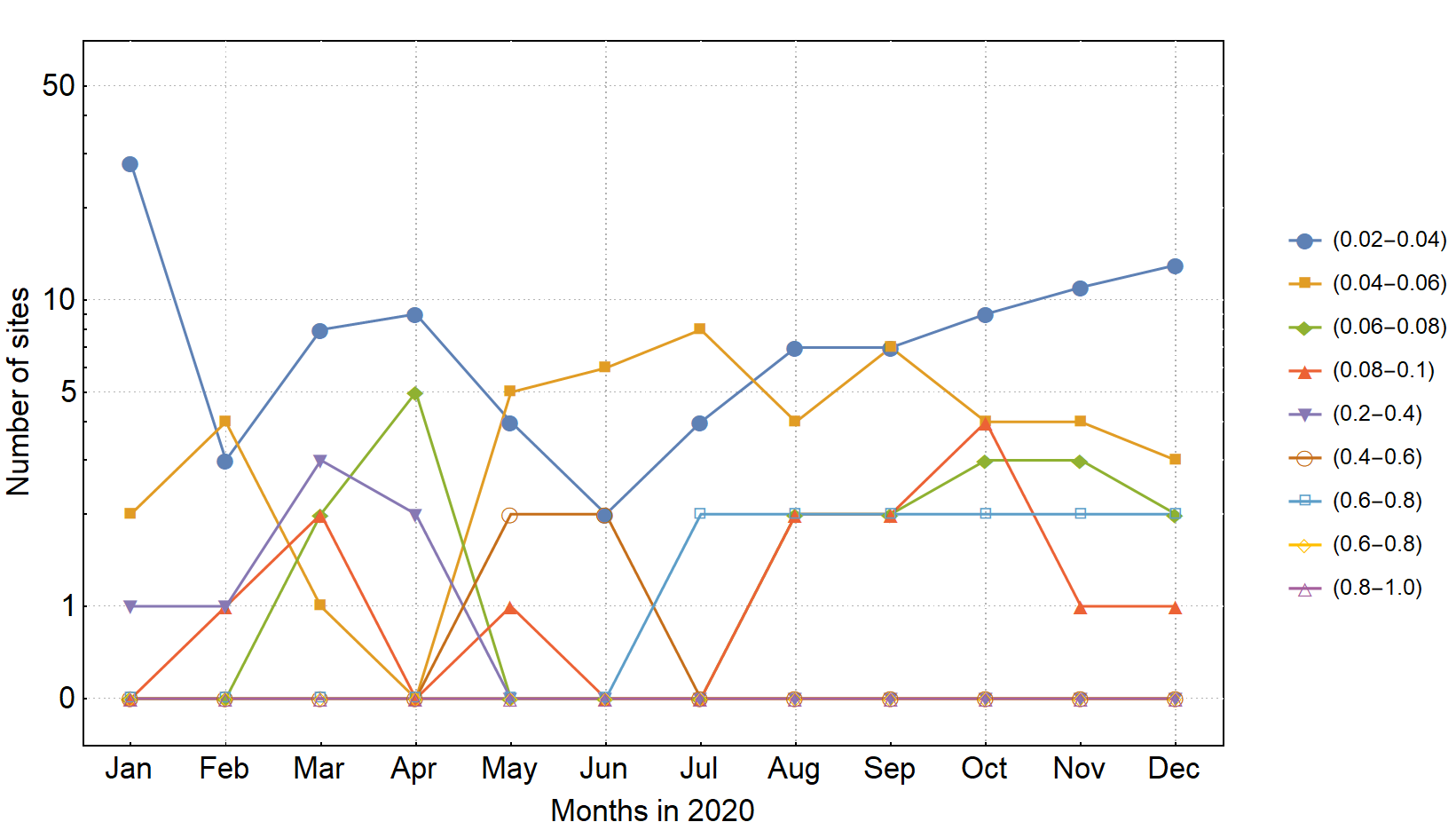 | 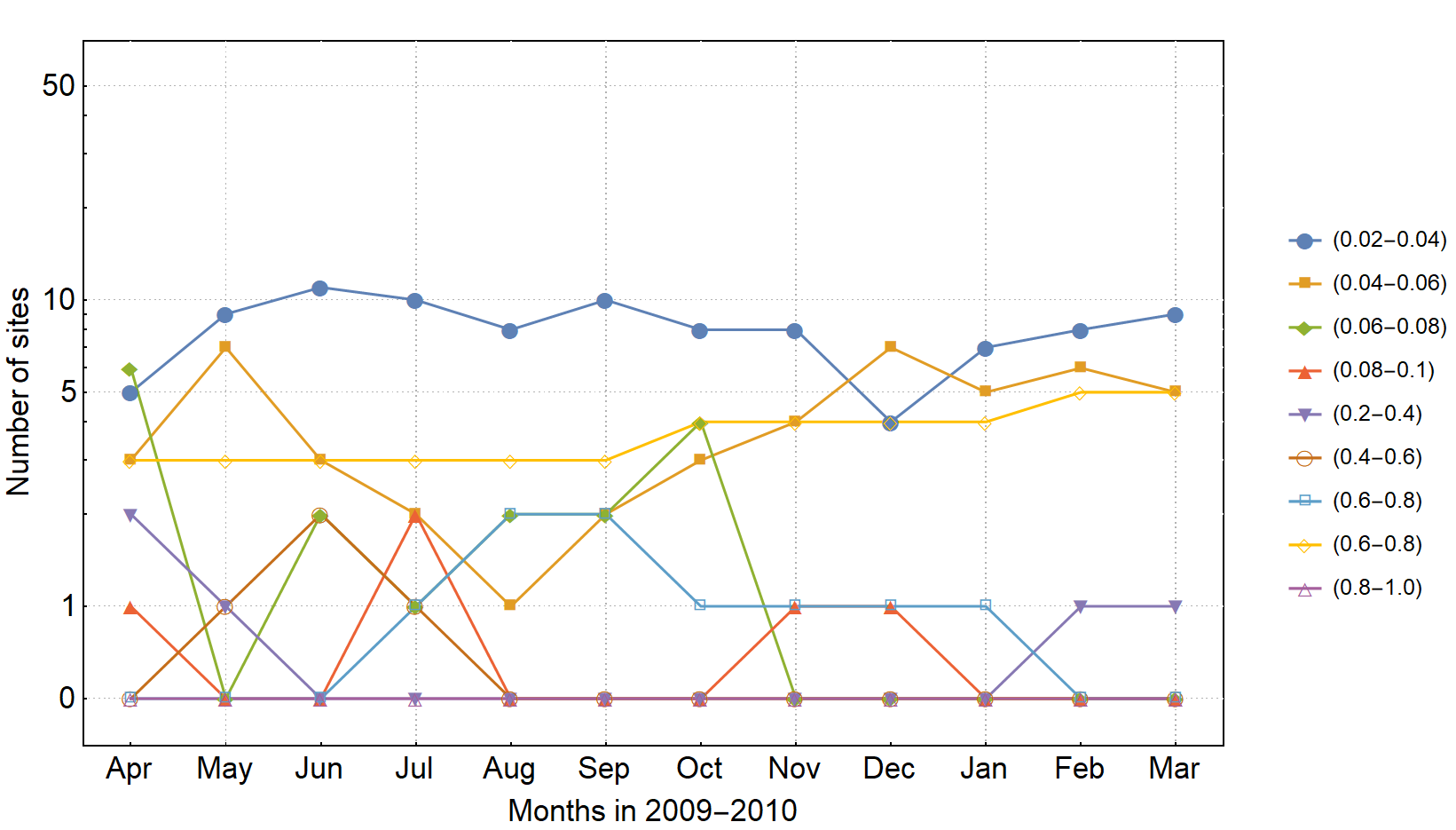 |
| **Figure S2: (A, B)** Site frequency spectrum and genetic distance (in the inset) over time for SARS-CoV-2 (left column) and pH1N1 (right column). By comparing the main and ancestral alignments, each site in the main alignment is defined as invariant if it is identical to the ancestral nucleotide, or variant if it is not. Number of sites are counted based on the number of differences between the ancestral and main nucleotide positions. **(C, D)** Number of new single-nucleotide differences (singletons) added to the dataset per month. **(E, F)** Number of polymorphic sites in each frequency range over time. | |

| **(A)** | **(B)** |
| --- | --- |
| 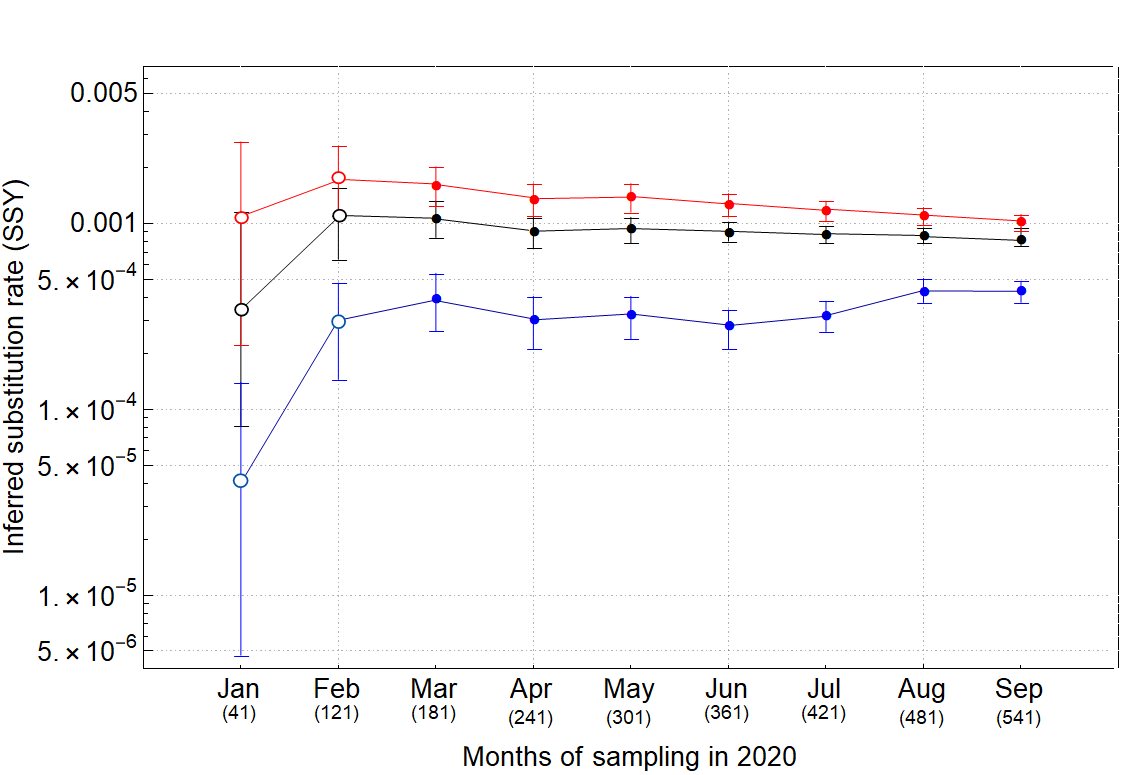 | 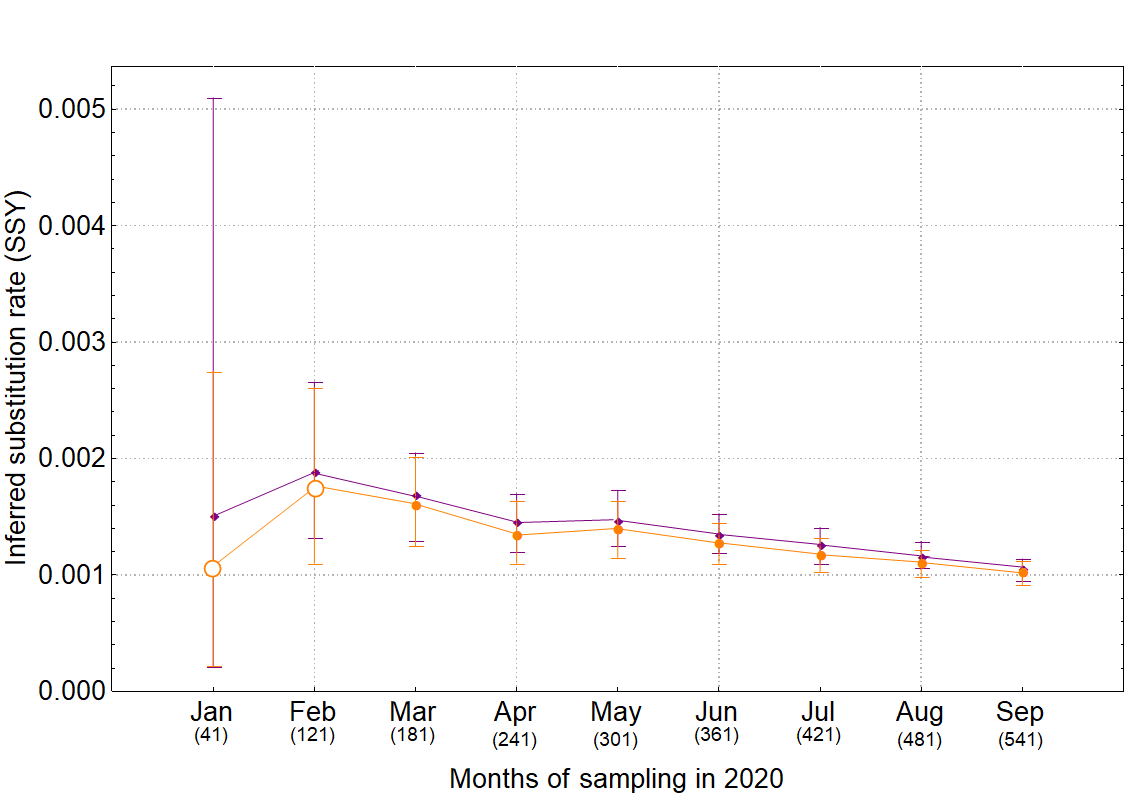 |
| **Figure S3**: **(A)** Mean clock rate (black), the rates at the terminal (red) and internal (blue) branches of SARS-CoV-2 alignments with masked sites. **(B)** Inferred substitution rate at terminal branches of SARS-CoV-2 alignments without (purple) and with (orange) masked sites. Open circles represent nonconvergence for at least one parameter in the Bayesian analysis. | |
